# Harmonic components of photoplethysmography and pathological patterns: A cross-sectional study

**DOI:** 10.1097/MD.0000000000034200

**Published:** 2023-09-01

**Authors:** Young-Jae Park, Jin-Moo Lee, Ka-Hye Choi

**Affiliations:** a Department of Biofunctional Medicine and Diagnostics, College of Korean Medicine, Kyung Hee University, Seoul, Republic of Korea; b Department of Diagnosis and Biofunctional Medicine, Kyung Hee University Hospital at Gangdong, Seoul, Republic of Korea; c Department of Biofunctional Medicine and Diagnostics, Graduate School, College of Korean Medicine, Kyung Hee University, Seoul, Republic of Korea; d Department of Gynecology, College of Korean Medicine, Kyung Hee University, Seoul, Republic of Korea; e Department of Women Health Clinic, Kyung Hee University Hospital at Gangdong, Seoul, Republic of Korea

**Keywords:** arterial stiffness, blood stasis, food retention, phlegm, second derivative of photoplethysmography, total harmonic distortion

## Abstract

This study aimed to examine whether the 3 harmonic components (HCs) of photoplethysmography (PTG) – total harmonic distortion (THD), harmonic power (HP), and normalized harmonic amplitude (HA) – have aging effects and may serve as an arterial stiffness marker and examine the relationship between HCs and clinical severity of pathological patterns. This study had a retrospective chart review design, and electronic medical records of 173 female patients (age: 38.57 ± 11.64 years) were reviewed. Patients were asked to complete the phlegm, blood stasis (BS), and food retention (FR) pattern questionnaires and underwent PTG and the second derivative of PTG measurements. THD, HP, and HA data were extracted till the 12th HCs from the raw PTG data. THD and HA had an aging effect (*β*: −0.179 to −0.278) and were related to *b/a (r*: −02.76 to −0.455) and *d/a (r*: 0.265–0.360) of the second derivative of PTG. In the younger group (≤33 years), HP and HA were positively correlated with phlegm, BS, and FR patterns (*r*: 0.257–0.370), while HP was positively correlated with the FR pattern (*r*: 0.278–0.315) in the middle age group (34–45 years). In the older group (≥46 years), HP and HA were positively or negatively correlated with the phlegm pattern (*r*: ±0.263 to ±0.440). HCs may serve as an arterial stiffness marker, and may be partially related to phlegm, BS, and FR patterns. Aging effect needs to be considered when utilizing HCs as an indicator of phlegm, BS, and FR patterns.

## 1. Introduction

Photoplethysmography (PTG) is a noninvasive device for detecting blood volume changes in living tissue by optical means.^[1]^ The PTG signal reflects blood movement in the vessel from the heart, spreading throughout the body’s peripheral capillaries in a wave-like motion.^[2]^ PTG can be easily obtained from the tissue pads of the ears, fingers, and toes where superficial vasculature is abundantly distributed.^[3]^ Although PTG estimates pulse waveform through blood volume changes on the peripheral artery, pressure wave estimates pulse waveform through arterial wall pressure changes detected using pressure sensor of tonometry or pulse diagnosis system. Pressure pulse wave has similarities to PTG, with similar changes occurring in vascular diseases such as damping and a loss of pulsatility.^[4]^ Owing to its convenience and cost-effectiveness, PTG has been widely utilized for estimating arterial stiffness, diagnosing atherosclerosis, respiratory monitoring and evaluating acute changes in peripheral arteries using mental stress.^[3,5]^ PTG or pressure waveform contour includes reflective components generated by reflection at the bifurcations of the blood vessels and returning to the center. Reflective components comprise tidal and dicrotic waves and dicrotic notch, and the peaks or notch of these components become blunted or dampened as arterial stiffness increases.^[4]^ As dampening of reflective components occurs frequently on a raw signal of PTG, it is difficult to estimate arterial stiffness severity from the raw PTG data. To overcome this limitation, a concept of the second derivative of the photoplethysmography (SDPTG) has been suggested.^[6]^ SDPTG indicates PTG indices extracted from double differentiation of raw PTG signal. Through double differentiation, reflective components may be revealed more clearly. Like raw PTG signal, SDPTG indices are associated with aging, emotional condition, quality of life, and arterial stiffness.^[7–9]^

Together with time-domain analysis such as SDPTG, frequency domain analysis of PTG has been suggested to describe the physiological and pathological conditions of blood supply.^[1]^ Among frequency domain analytic methods, harmonic analysis (HA) aims to decompose the arterial pulse wave into harmonic components (HCs) using Fourier transformation.^[10]^ PTG contour is not a pure sinusoid wave but a semi-periodic signal comprising waves with diverse frequencies.^[11]^ PTG components comprising waves with diverse frequencies can be transformed to an array of power or amplitude components of HCs, where the frequency of each harmonic is located among the integer multiples of the fundamental frequency. Fundamental frequency (first HC of the PTG) denotes the heart rate per minute. From an electrical point of view, ideal alternating current is purely sinusoidal, and there may have none of the harmonics. However, an electrical circuit comprises diverse sub-parts including rectifiers, power transistors, converters, and power supplies, and the complex circuit may result in a non-sinusoidal current.^[12]^ Non-sinusoidal current may include HCs, which are unwanted higher frequencies and impede the flow of alternating current.^[12]^ The sum of all HCs is defined as total harmonic disturbance (THD), and like the HC concept, minimal THD denotes the flow of alternating current with minimal impeding factor.^[13]^ Arterial wall pulse waveform is a semi-periodic or non-sinusoidal wave to which diverse factors, including waveform differences in systolic and diastolic stages and adding reflective components to the original pulse waveform, may contribute. Earlier research on HA of PTG or pressure wave reported that HCs had aging effect, similar to the time-domain analysis indices. Sherebrin and Sherebrin^[1]^ reported that the second harmonic power (HP) was greater in the young group than in the older group, speculating that this difference was associated with the disappearance of the dicrotic notch in the older group. Wang et al^[14]^ reported that normalized amplitudes of the third and fourth HCs decreased with aging. Recently, Wu et al reported that the THD of the PTG showed satisfactory intra-subject reliability and was associated with the risk factors of diabetes in type 2 diabetic patients along with waist circumstance.^[15]^

Some studies suggested that the properties of the large artery supplying organs were reflected on the specific HC of the pressure wave. For example, the second and third HCs of patients with myocardial infarction decreased in the acute stage, and as the patients recovered, the HCs gradually increased.^[16]^ Ligation of renal and spleen arteries reduced the second and third HCs, respectively, in an animal study.^[17]^ A study reported that first HC may indicate liver dysfunction as identified via blood testing.^[18]^ Finally, combining the studies on the relationship between HCs and internal organs with the East-Asian medicine theory, Lin Wang et al^[19]^ speculated the concept of “organ–frequency relation” where the summed HCs, and first, second, third, and fourth HCs may reflect the properties of the heart, liver, kidney, spleen, and lung, respectively. According to Wang’s speculation, a study interpreted the decrease in the third HC of pulse waveform in atopic dermatitis as dysfunction of the spleen.^[20]^

Considering the aforementioned study results of HA, the clinical utility of HCs may be summarized in 2 ways – an arterial stiffness marker and indicator of internal organ condition. However, these 2 characteristics are not consistent with each other. For example, a study reported that the second, third, fourth, fifth, and sixth HCs were related to the SDPTG indices, implying that HCs generally reflected the arterial stiffness.^[21]^ However, Wang et al assigned a specific HC to a specific internal organ, and this meant that “organ–frequency relation” was local and not general. Moreover, from the East-Asian medicine point of view, dysfunction of internal organ should be evaluated through identification of pathological pattern (PP) before anatomical or disease characteristics. PP is defined as a determined pattern or syndrome through the comprehensive analysis of symptoms and signs, referring to the cause, nature, and location of the illness.^[22]^ For example, phlegm pattern denotes a pathological condition manifesting with sputum, dizziness, thick tongue coating, and bead-like pulse. The formation of phlegm, blood stasis (BS), and food retention (FR) patterns is associated with dysfunction of the spleen and lungs,^[23]^ liver and heart,^[24]^ and spleen and stomach,^[25]^ respectively. Phlegm, BS, and FR patterns are considered factors impeding the flow of qi, blood, and fluid.^[23–25]^ If HCs are related to internal organs, and PPs denote dysfunction of internal organs, HCs may be related to the clinical severity of phlegm, BS, and FR patterns. Therefore, this study aimed to examine whether the aging effect and the relationship between HC and SDPTG indices were consistent with those reported previously,^[21]^ and examine the relationship between HCs and the clinical severity of phlegm, BS, and FR patterns. The clinical severity of PP was estimated using the Phlegm Pattern Questionnaire (PPQ), BS Questionnaire (BSQ), and FR Questionnaire (FRQ).^[23–25]^ In addition to the examination of the relationship between HCs and clinical severity of PPs, we examined whether the relationships were locally limited to a specific HC or internal organ or generally distributed to most of the HCs and internal organs.

Another purpose of this study was to compare 3 HCs of PTG – normalized harmonic amplitude (HA), HP, and THD. As HA is a frequency domain analysis, the analysis results are primarily derived in decibel, a relative power unit. Power values are then transformed to millivolt, an amplitude unit. Almost all previous studies reported their results using normalized HA units, and few studies addressed which measurement units among HP, HA, and THD were more indicative of aging effect or dysfunction of internal organs. If the THD of pulse waveform reflects total impediment of blood circulation similarly to an electrical circuit, it may serve as a strong arterial stiffness marker or indicator of internal organs.

In this study, the aging effect of HCs and the relationship among HCs, SDPTG indices, and dysfunction of internal organs estimated using PPQ, BSQ, and FRQ scores were examined using HP, normalized HA, and THD values.

## 2. Materials and methods

### 2.1. Subjects

This study had an observational and cross-sectional study design. Electronic medical records of 173 female patients (mean age, 38.57 ± 11.64 years) were reviewed. The outpatients visited the Women’s Health Clinic of Kyung Hee University Medical Hospital at Gangdong with menstrual dysfunction, infertility, climacteric syndrome, post-operative management, and post-labor management. They were asked to complete the PPQ, BSQ, and FRQ. In addition to completing the 3 pattern questionnaires, they underwent PTG and SDPTG measurements. The study protocol was approved by the Institutional Review Board of the Kyung Hee University Medical Hospital at Gangdong.

### 2.2. Mewasurements

#### 2.2.1. PPQ, BSQ, and FRQ.

**Figure 1.**
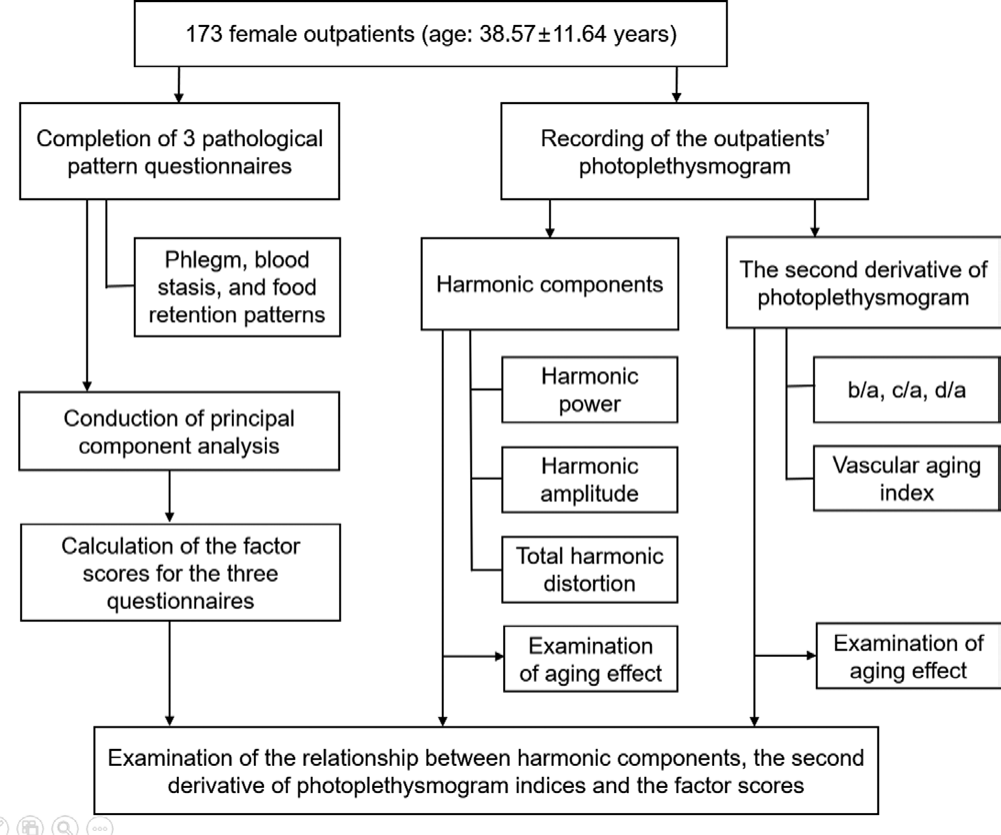
An entire flow of measurements and data analysis of this study.

Figure 1 depicts the entire flow of measurements and data analysis in this study. Phlegm is a viscous, turbid pathological product that accumulates in the body, leading to diverse respiratory, neurological, and gastrointestinal problems, including nasal discharge, sputum, dizziness, palpitation, ingestion, and mucousy stool.^[23]^ The PPQ comprises 25 phlegm-related items. Blood stasis refers to retarded blood flow, blood varicose, or clotting that is the consequence of bleeding, trauma, or a lump or tumor.^[24]^ The BSQ comprises 12 BS-related items. FR is a pathological condition, manifesting as epigastric or abdominal fullness or pain, dyspepsia, water brash, sickness, languidness, edema, weight gain, or joint pain.^[25]^ The FRQ comprises 17 FR-related items. The PPQ, BSQ, and FRQ are rated on a 7-point Likert scale (1 = disagree very strongly and 7 = agree very strongly).

#### 2.2.2. HC measurements.

Each subject rested for 10 minutes on a comfortable chair. PTG signals were recorded for 300-s periods on the index finger of the left hand using MP100 system (BIOPAC Systems, Inc., Goleta, CA). Among the overall PTG data, the 60-seconds period between 30- and 90-seconds periods was considered because it has a stable PTG signal, and its PTG signal peaks and baselines often fluctuate after 180 seconds due to respiration or motion artifact. Cropped PTG data were imported to MATLAB software (MathWorks, Natick, MA), and THD, HP, and normalized HA were calculated. HP was calculated using “periodogram (data, rectwin (N), N, Fs)” syntax of the MATLAB, where data indicates the above-mentioned 60-seconds PTG data, N indicates the length of the data, rectwin (N) indicates returning of a rectangular window of length N in a column vector, and Fs indicates sampling frequency. The peridiogram for a sequence [x1, … , xn] is given by following formula $S\left(e^{\;jw}\right)=\frac{1}{n}{\left|\overset{n}{\underset{l=1}{\sum }}x_{l}e^{\;-jwl}\right|}^{2}$ and HP is defined as $S\left(e^{\;jw}\right)/Fs$.^[26]^ In this study, 12 HPs were extracted. For example, H_1_ and H_7_ meant the power of the first and seventh HCs, respectively. In terms of THD, THD_2_ meant the ratio of summation of the first and second HCs and the first HC, while THD_5_ meant the summation of the first, second, third, fourth, and fifth HCs and the first HC. As THD_1_ equals to HP_1_, 11 THD (THD_2_ – THD_12_) were extracted. Normalized HA was defined as the nth HA divided by the first HA. For example, AH_9_/AH_1_ meant the ratio of the ninth and first HAs. The amplitude of the HCs was calculated from the HP using the formula $Amplitude\;of\;the\;nth\;harmonic\;component\;\left(volts\right)=HP1*10^{\left(\frac{HPn}{10}\right)}$ (HP_1_, the first harmonic power; HP_*n*_, *n*th harmonic power).^[27]^ In this study, 12 HPs (HP_1_ – HP_12_) and 11 normalized HAs (AH_2_/AH_1_ – AH_12_/AH_1_) were calculated.

#### 2.2.3. SDPTG measurements.

After recording the PTG signal to calculate harmonic components, another PTG sensor was attached to the index finger of the subjects’ left hand using the SA-3000P (Medicore Co., Seoul, South Korea). SDPTG comprises 4 systolic waves (*a, b, c*, and *d*), which correspond to the systolic stage of the heart and a diastolic wave (*e*), which corresponds to the diastolic stage of the heart.^[7]^ SA-3000P recorded 180-s PTG data and automatically calculated the relative amplitudes of 3 SDPTG indices – *b/a, c/a*, and *d/a*. Using the 4 indices, vascular aging index (VAI = (*b–c–d*)/*a*) was calculated.^[2]^ In this study, *e/a* was not considered because SA-3000P did not present information on the *e* wave.

### 2.3. Data analysis

Simple regression models for HCs and SDPTG indices, with age as an independent variable, were used to examine the effects of aging on the variables. If there was an aging effect on the HCs and the SDPTG indices, the following analyses were performed separately in 3 age groups (younger, middle age, and older groups). Two cutoff points corresponding to 1/3 and 2/3 years of all age distribution were determined, and the subjects below the 1/3 points, above 2/3 points, and between 1/3 and 2/3 points, were categorized into the younger, older, and middle age groups, respectively. Principal component analysis followed by varimax rotation was performed to examine dimensionality and extract factors of the PPQ, BSQ, and FRQ. On condition that there was aging effect, Pearson’s correlation analyses between HC and SDPTG indices were performed separately in the 3 age groups. Finally, Pearson’s correlation analyses between HCs and the factor scores of the PPQ, BSQ, and FRQ were performed separately in the 3 age groups. In correlation analysis, a correlation coefficient of > 0.7 was considered a “very strong correlation,” 0.3 to 0.7 was considered a “strong correlation,” and 0.1 to 0.3 was considered a “weak correlation.”^[28]^ In this study, statistical analyses were performed using Statistical Package for Social Sciences version 21 (SPSS, Inc., Chicago, IL). Values were presented as means ± standard deviations, and *P* < .05 was considered statistically significant.

## 3. Results

**Figure 2.**
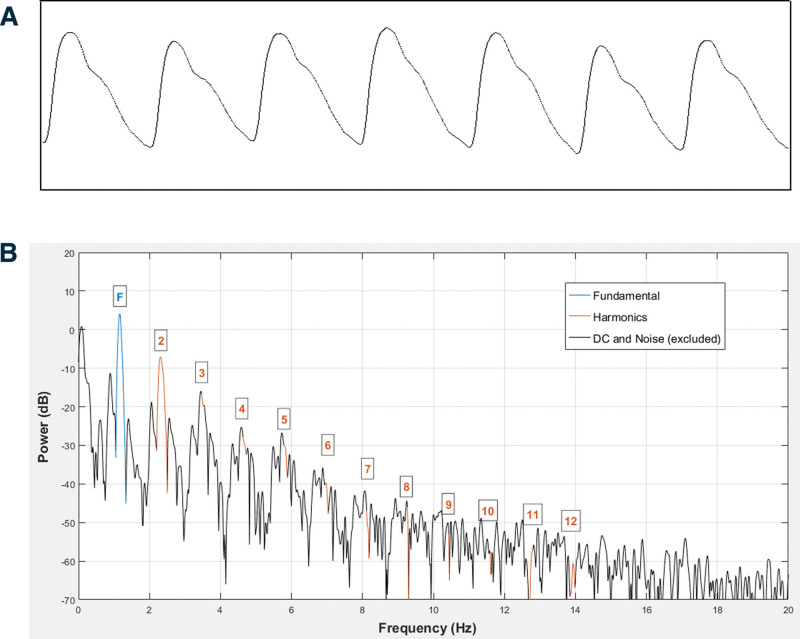
A sample of raw photoplethysmography signal and visualization of power of the 12 harmonic components. A; raw photoplethysmography data, B; power of the 12 harmonic components. DC = direct current.

Table 1 lists the descriptive characteristics of 3 questionnaire scores, HCs, and SDPTG indices. Figure 2 shows a sample of the raw PTG signal and the harmonic powers of 12 HCs. Table 2 shows the simple regression analysis results for SDPTG and HC indices with age as an independent variable. The standardized beta (*β*) values of SDPTG indices were 0.524 to 0.644, indicating that age independently and dominantly affected SDPTG indices. The *β* values of the 11 THD indices were −0.179 to −0.202, indicating that THD indices had an aging effect. However, *P* values of the normalized HAs were significant only for AH_2_/AH_1_, AH_3_/AH_1_, AH_4_/AH_1_, and AH_5_/AH_1_ (*β* values; −0.205 to −0.278), and the other HAs did not show an aging effect. In terms of HP indices, only the fourth HC showed an aging effect (*β* values = −0.294).

**Table 1:** Descriptive statistics of pattern questionnaire scores, and the second derivative and harmonic components of photoplethysmography.

Scale	Subscale	Minimum	Maximum	Mean ± SD	Scale	Subscale	Minimum	Maximum	Mean ± SD
Pattern questionnaire (Scores)	Phlegm	31.00	123.00	78.34 ± 19.77	THD (dB)	THD_2_	−19.08	−4.52	−9.73 ± 2.09
	Blood stasis	12.00	56.00	31.85 ± 9.92		THD_3_	−18.36	−3.56	−9.18 ± 2.17
	Food retention	22.00	83.00	51.36 ± 13.14		THD_4_	−18.26	−3.45	−9.11 ± 2.18
SDPTG (ratio)	VAI	−145.34	125.82	−54.82 ± 37.44		THD_5_	−18.19	−3.35	−9.07 ± 2.18
	*b/a*	−107.40	22.10	−71.88 ± 15.60		THD_6_	−18.19	−3.35	−9.06 ± 2.18
	*c/a*	−48.42	26.50	−8.21 ± 13.12		THD_7_	−18.18	−3.35	−9.06 ± 2.18
	*d/a*	−91.44	−4.62	−28.43 ± 12.36		THD_8_	−18.18	−3.35	−9.06 ± 2.18
Frequency (Hz)	Fundamental frequency	0.08	1.52	1.14 ± 0.18		THD_9_	−18.18	−3.35	−9.06 ± 2.18
Harmonic power (dB)	1st HP	−7.74	13.91	5.82 ± 4.22		THD_10_	−18.18	−3.35	−9.06 ± 2.18
	2nd HP	−17.30	4.24	−3.91 ± 4.85		THD_11_	−18.18	−3.35	−9.05 ± 2.18
	3rd HP	−32.73	−2.93	−13.17 ± 5.92		THD_12_	−18.18	−3.35	−9.04 ± 2.20
	4th HP	−47.31	−11.34	−22.98 ± 7.24	Normalized harmonic amplitude (ratio)	AH_2_/AH_1_	1.24E-02	0.35	0.12 ± 0.05
	5th HP	−45.23	−13.09	−26.34 ± 7.01		AH_3_/AH_1_	3.89E-04	0.09	0.02 ± 0.01
	6th HP	−58.30	−17.60	−33.16 ± 7.57		AH_4_/AH_1_	8.86E-06	0.01	2.35E-03 ± 2.34E-03
	7th HP	−64.89	−25.12	−40.76 ± 7.92		AH_5_/AH_1_	7.21E-06	9.87E-03	1.13E-03 ± 1.27E-03
	8th HP	−67.09	−28.82	−45.76 ± 7.70		AH_6_/AH_1_	1.69E-06	4.90E-03	2.96E-04 ± 4.82E-04
	9th HP	−70.47	−30.16	−50.16 ± 8.03		AH_7_/AH_1_	7.00E-08	9.64E-04	6.20E-05 ± 1.05E-04
	10th HP	−73.61	−11.09	−54.80 ± 8.63		AH_8_/AH_1_	1.65E-08	6.10E-04	2.57E-05 ± 6.36E-05
	11th HP	−78.27	−17.96	−57.80 ± 8.60		AH_9_/AH_1_	5.99E-09	3.85E-04	1.16E-05 ± 4.10E-05
	12th HP	−76.29	−3.42	−59.62 ± 8.29		AH_10_/AH_1_	1.03E-08	0.03	2.03E-04 ± 2.38E-03
						AH_11_/AH_1_	2.90E-09	6.40E-04	4.83E-05 ± 5.01E-04
						AH_12_/AH_1_	8.16E-09	0.18	1.06E-03 ± 1.38E-02

AH = amplitude of harmonics, HP = harmonic power, SDPTG = second derivative of the photoplethysmography, THD = total harmonic distortion, THD = total harmonic distortion, VAI = vascular aging index.

**Table 2:** Simple regression analyses for SDPTG and harmonic components with age as an independent variable.

Scale	Subscale	*β*	*t* value	*P* value	Scale	Subscale	*β*	*t* value	*P* value
SDPTG (ratio)	VAI	0.644	11.001	**<.001**	THD (dB)	THD_5_	−0.194	−2.588	**.010**
	b/a	0.625	10.466	**<.001**		THD_6_	−0.195	−2.601	**.010**
	c/a	−0.524	−8.037	**<.001**		THD_7_	−0.195	−2.603	**.010**
	d/a	−0.638	−10.823	**<.001**		THD_8_	−0.195	−2.604	**.010**
Power of harmonic component (dB)	1st HP	0.041	0.538	.591		THD_9_	−0.195	−2.604	**.010**
	2nd HP	−0.041	−0.542	.588		THD_10_	−0.197	−2.629	**.009**
	3rd HP	−0.078	−1.026	.306		THD_11_	−0.197	−2.633	**.009**
	4th HP	−0.294	−4.030	**<.001**		THD_12_	−0.202	−2.693	**.008**
	5th HP	−0.145	−1.912	.058	Normalized harmonic amplitude (ratio)	AH_2_/AH_1_	−0.269	−3.656	**<.001**
	6th HP	−0.040	−0.527	.599		AH_3_/AH_1_	−0.242	−3.263	**.001**
	7th HP	−0.081	−1.061	.290		AH_4_/AH_1_	−0.278	−3.787	**<.001**
	8th HP	−0.099	−1.299	.196		AH_5_/AH_1_	−0.205	−2.745	**.007**
	9th HP	−0.046	−0.601	.549		AH_6_/AH_1_	0.011	0.147	.883
	10th HP	−0.026	−0.337	.737		AH_7_/AH_1_	−0.022	−0.292	.771
	11th HP	−0.131	−1.730	.085		AH_8_/AH_1_	−0.056	−0.728	.467
	12th HP	−0.098	−1.292	.198		AH_9_/AH_1_	−0.022	−0.286	.775
Harmonic frequency (Hz)	Fundamental frequency	−0.086	−1.132	.259		AH_10_/AH_1_	−0.054	−0.709	.480
THD (dB)	THD_2_	−0.179	−2.381	**.018**		AH_11_/AH_1_	−0.048	−0.627	.531
	THD_3_	−0.185	−2.458	**.015**		AH_12_/AH_1_	−0.058	−0.766	.445
	THD_4_	−0.191	−2.539	**.012**					

Bold letters indicate significant *P* value.

AH = amplitude of harmonics, HP = harmonic power, SDPTG = second derivative of the photoplethysmography, THD = total harmonic distortion, VAI = vascular aging index.

Table 3 lists the principal component analysis results of the PPQ. Six factors were extracted, and the total percentage of the variance was 61.17%. Factors 1 and 2 comprised gastrointestinal problem-related items (PP1; gastrointestinal factor) and psychological problem-related items (PP2, psychological factor), respectively. Factors 3 and 4 comprised neurological problem-related items (PP3, neurological factor). Table 4 lists the principal component analysis results of the BSQ and the FRQ. In terms of the BSQ, factors 1 and 2 comprised pain–bruising-related items (BS1, pain–bruising factor) and dark-blue sign-related items (BS2, dark-blue factor). Factor 3 comprised traumatic experience-related items (BS3, trauma factor). In terms of the FRQ, factors 1, 2, 3, and 4 comprised upset stomach-related items (FR1, upset stomach factor), edema–weight gain-related items (FR2, edema–weight gain factor), eating-related response items (FR3, eating response factor), and food allergy-related items (FR4, food allergy factor), respectively.

**Table 3:** Principal component analysis results of the phlegm pattern questionnaire items.

Item	Factor					
	PP1	PP2	PP3	PP4	PP5	PP6
Stomach fullness	**0.750**	0.189	0.066	0.226	0.215	0.086
Indigestion	**0.711**	0.181	0.290	0.081	0.012	0.179
Sickness	**0.657**	0.142	0.261	0.180	0.110	0.0154
Abdominal rumbling	**0.613**	0.101	0.0140	−0.099	0.317	−0.098
Mucousy stool	**0.402**	0.112	0.136	0.178	0.289	0.112
Palpitations	0.213	**0.792**	0.131	0.178	0.033	0.196
Startled by noise	0.107	**0.775**	0.258	0.006	0.109	0.0074
Chest discomfort	0.288	**0.721**	0.170	0.136	0.230	0.159
Breath shortness	0.511	**0.521**	0.003	0.281	0.105	0.205
Flank pain	0.020	**0.486**	0.186	0.190	0.431	−0.046
Headache	0.308	0.199	**0.781**	0.038	0.117	0.012
Dizziness	0.238	0.214	**0.699**	−0.062	0.158	0.259
Head unclearness	0.380	0.310	**0.603**	0.049	0.015	0.297
Itching	−0.071	0.101	**0.576**	0.270	0.355	0.155
Sputum	0.107	0.059	−0.047	**0.832**	0.004	0.185
Cough	0.161	0.180	0.040	**0.788**	0.001	−0.058
Feeling of foreign body	0.090	0.063	0.113	**0.723**	0.203	−0.040
Tinnitus	0.165	0.210	0.055	0.013	**0.681**	0.188
Dark circle	0.293	−0.014	0.098	−0.024	**0.610**	0.225
Lumps	0.120	0.170	0.145	0.170	**0.598**	0.061
Joint pain	−0.095	0.154	0.192	0.060	0.180	**0.645**
Limb heaviness	0.219	0.217	0.356	0.042	0.053	**0.641**
Poor appetite	0.459	0.088	−0.135	0.064	0.093	**0.575**
Yellow face	0.189	−0.032	0.128	0.028	0.452	**0.547**
Fatigue	0.151	0.395	0.370	−0.142	0.190	**0.448**
Variance explained (%)	12.95	11.63	10.09	9.09	8.76	8.66

Bold numbers indicate the greatest factor loading among the 6 factors and their corresponding items.

PP = phlegm pattern.

**Table 4:** Principal component analysis of blood stasis and food retention patterns.

Item	Blood stasis			Item	Food retention			
	BS1	BS2	BS3		FR1	FR2	FR3	FR4
Joint pain	**0.771**	0.063	0.125	Abdominal fullness	**0.810**	0.138	0.141	0.130
Night pain	**0.733**	0.148	0.147	Upper abdominal pain	**0.736**	0.117	0.113	0.135
Prolonged dull pain	**0.700**	0.140	0.018	Sickness	**0.667**	0.192	0.282	0.054
Flank pain	**0.621**	0.230	0.086	Water brash	**0.617**	0.298	0.252	−0.011
Frequent bruising	**0.547**	0.120	0.300	Frequent upset stomach	**0.557**	0.238	−0.021	0.388
Dark circle	0.001	**0.827**	0.047	Edema	0.221	**0.647**	0.054	0.294
Dark-blue tongue	0.158	**0.826**	0.176	Frequent urination	0.106	**0.634**	0.275	−0.071
Tarry stool	0.155	**0.537**	0.277	Joint pain	0.195	**0.548**	−0.001	0.365
Abdominal lump	0.320	**0.519**	0.017	Weight gain	0.121	**0.544**	0.004	0.136
Abdominal pain	0.465	**0.506**	−0.020	Feeling languid after eating	0.428	**0.532**	0.095	0.137
Recent traffic accident	0.054	0.126	**0.844**	Frequent belching	0.020	**0.482**	0.464	−0.104
Recent sprain	0.244	0.124	**0.766**	Feeling heavy in the body	0.356	**0.462**	0.155	−0.169
				Bowel movement after eating	0.133	0.017	**0.758**	0.119
				Stomach pain after eating	0.318	0.127	**0.722**	0.162
			Mucousy stool	0.136	0.134	**0.670**	0.063
				Water change−related abdominal pain	0.127	0.110	0.258	**0.791**
				Food allergy	0.099	0.081	0.037	**0.776**
Variance explained (%)	22.80	19.37	12.88	Variance explained (%)	17.19	14.20	12.45	10.36

Bold numbers indicate the greatest factor loading among the factors of blood stasis, and food retention questionnaires.

BS = blood stasis, FR = food retention.

As the 1/3 and 2/3 points of the total age distribution were 33 and 45.9 years, women aged ≤ 33 years, ≥46 years, and between 34 and 45 years were categorized into the young group (n = 59), old group (n = 58), and middle age group (n = 56), respectively. Table 5 lists Pearson’s correlations between HCs and SDPTG indices in the 3 age groups. In middle age group, all THD indices generally had strong or weak negative correlations with *b/a* index (*r*: −0.276 to −0.313) and weak positive correlations with *d/a* index (*r*: 0.268–0.292). Normalized HAs had strong or weak negative correlations with VAI (*r*: −0.292 to −0.321) and *b/a* index (*r*: −0.300 to −0.392) and strong or weak positive correlations with *d/a (r*: 0.276–0.360), limitedly to AH_2_/AH_1_, AH_3_/AH_1_, AH_4_/AH_1_, AH_5_/AH_1_, and AH_10_/AH_1_. In the older group, THD indices did not show any significant correlations with SDPTG indices. Instead, the fourth, seventh, eighth, ninth, 11th, and 12th HPs had negative correlations with VAI (*r*: −0.303 to −0.372) and *b/a* index (*r*: −0.264 to −0.455) and positive correlations with *d/a* index (*r*: 0.259–0.375). Similar to the middle age group, in the old group, normalized HAs had negative correlations with VAI (*r*: −0.273 to −0.404) and *b/a* index (*r*: −0.319 to −0.367) and positive correlations with *d/a* index (*r*: 0.265–0.339), limitedly to AH_3_/AH_1_, AH_4_/AH_1_, AH_5_/AH_1_, and AH_7_/AH_1_. Fundamental frequency had strong negative correlations with *c/a* index (*r* = −0.370). In the younger group, only AH_2_/AH_1_ had a weak negative correlation with VAI (*r* = −0.299) and a strong positive correlation with *d/a* index (*r* = 0.353).

**Table 5:** Correlations between harmonic components and second derivative indexes according to age.

Age group		Younger (n = 59)				Middle age (n = 56)				Older (n = 58)			
Scale	Subscale	VAI	*b/a*	*c/a*	*d/a*	VAI	*b/a*	*c/a*	*d/a*	VAI	*b/a*	*c/a*	*d/a*
Total harmonic distortion	THD_2_	−0.178	−0.155	0.031	0.199	−0.189	**−0.276** *	−0.081	**0.268** *	−0.192	−0.202	0.031	0.206
	THD_3_	−0.178	−0.155	0.035	0.187	−0.216	**−0.305** *	−0.063	**0.289** *	−0.225	−0.227	0.064	0.213
	THD_4_	−0.176	−0.153	0.033	0.187	−0.222	**−0.309** *	−0.053	**0.291** *	−0.232	−0.232	0.070	0.218
	THD_5_	−0.178	−0.155	0.034	0.189	−0.226	**−0.313** *	−0.049	**0.292** *	−0.235	−0.234	0.074	0.220
	THD_6_	−0.179	−0.156	0.035	0.190	−0.225	**−0.313** *	−0.050	**0.291** *	−0.236	−0.235	0.077	0.220
	THD_7_	−0.179	−0.156	0.036	0.190	−0.226	**−0.313** *	−0.049	**0.291** *	−0.237	−0.235	0.077	0.220
	THD_8_	−0.179	−0.156	0.036	0.190	−0.226	**−0.313** *	−0.049	**0.292** *	−0.237	−0.235	0.078	0.220
	THD_9_	−0.180	−0.156	0.036	0.190	−0.226	**−0.313** *	−0.049	**0.292** *	−0.237	−0.236	0.078	0.220
	THD_10_	−0.181	−0.158	0.041	0.192	−0.226	**−0.313** *	−0.049	**0.292** *	−0.237	−0.236	0.078	0.220
	THD_11_	−0.181	−0.158	0.042	0.192	−0.226	**−0.313** *	−0.049	**0.292** *	−0.237	−0.236	0.078	0.220
	THD_12_	−0.182	−0.160	0.057	0.193	−0.226	**−0.313** *	−0.049	**0.292** *	−0.237	−0.236	0.079	0.220
Harmonic frequency	Fundamental frequency	0.009	−0.017	−0.236	0.132	−0.109	−0.086	0.015	0.215	0.089	−0.037	**−0.370** **	0.016
Harmonic power	1st HP	0.231	0.156	−0.165	−0.166	0.229	0.144	−0.255	**−0.309** *	0.009	−0.168	−0.244	0.042
	2nd HP	0.104	0.052	−0.124	−0.038	0.158	0.052	**−0.265** *	−0.210	−0.074	−0.225	−0.190	0.122
	3rd HP	0.070	0.024	−0.088	−0.065	0.041	−0.067	−0.183	−0.100	−0.142	−0.236	−0.074	0.110
	4th HP	0.167	0.130	−0.172	−0.089	−0.063	−0.143	−0.090	0.058	**−0.372** **	**−0.455** **	−0.013	**0.375** **
	5th HP	0.108	0.080	−0.071	−0.115	−0.005	−0.078	−0.104	−0.049	−0.190	−0.226	0.081	0.142
	6th HP	0.189	0.161	−0.106	−0.173	0.032	−0.058	−0.101	−0.092	−0.190	−0.251	0.033	0.102
	7th HP	0.048	0.041	−0.018	−0.051	−0.089	−0.176	0.033	0.013	**−0.349** **	**−0.424** **	0.045	**0.286** *
	8th HP	0.109	0.128	−0.036	−0.103	−0.138	−0.182	0.092	0.067	**−0.303** *	**−0.331** *	0.191	**0.259** *
	9th HP	0.103	0.124	0.008	−0.116	−0.079	−0.168	0.030	−0.008	−0.210	**−0.264** *	0.154	0.219
	10th HP	−0.085	−0.088	0.201	−0.031	−0.137	−0.207	0.075	0.060	−0.225	−0.243	0.176	0.202
	11th HP	−0.126	−0.106	0.235	0.015	0.022	−0.031	−0.077	−0.029	−0.157	−0.122	**0.343** **	0.024
	12th HP	−0.081	−0.043	0.155	0.092	−0.129	−0.135	0.125	0.077	−0.230	−0.169	**0.422** **	0.141
Normalized harmonic amplitude	AH_2_/AH_1_	**−0.299** *	−0.237	0.060	**0.353** **	−0.224	**−0.300** *	−0.056	**0.310** *	−0.228	−0.238	0.046	0.257
	AH_3_/AH_1_	−0.236	−0.181	0.115	0.193	**−0.321** *	**−0.392** **	0.052	**0.360** **	**−0.346** **	**−0.319** *	0.192	0.216
	AH_4_/AH_1_	−0.049	0.007	−0.053	0.119	**−0.300** *	**−0.318** *	0.170	**0.276** *	**−0.404** **	**−0.367** **	**0.280** *	**0.339** **
	AH_5_/AH_1_	−0.087	−0.029	0.010	0.067	**−0.292** *	**−0.334** *	0.151	0.185	**−0.394** **	**−0.329** *	**0.387** **	**0.265** *
	AH_6_/AH_1_	0.069	0.069	−0.010	−0.105	−0.055	−0.141	−0.091	0.011	−0.231	−0.174	**0.314** *	0.130
	AH_7_/AH_1_	0.015	0.022	0.051	−0.040	−0.132	−0.223	0.048	−0.008	**−0.273** *	−0.215	**0.333** *	0.186
	AH_8_/AH_1_	−0.034	−0.042	0.139	−0.041	−0.154	−0.188	0.122	0.044	−0.211	−0.160	**0.263** *	0.173
	AH_9_/AH_1_	−0.040	−0.044	0.133	0.018	−0.183	−0.237	0.157	0.087	−0.198	−0.146	**0.275** *	0.151
	AH_10_/AH_1_	−0.027	−0.034	0.099	0.026	**−0.264** *	**−0.323** *	0.239	0.055	−0.108	−0.068	0.159	0.095
	AH_11_/AH_1_	−0.027	−0.034	0.100	0.026	−0.182	−0.181	0.218	0.065	−0.108	−0.068	0.160	0.094
	AH_12_/AH_1_	−0.027	−0.034	0.099	0.026	−0.142	−0.138	0.159	0.094	−0.107	−0.068	0.158	0.095

Bold letters indicated significant *P* value.

AH = amplitude of harmonics, HP = harmonic power, THD = total harmonic distortion, VAI = vascular aging index.

* *P* < .05.

** *P* < .01.

Tables 6–8 show Pearson’s correlations between HCs and factor scores of the PPQ, BSQ, and FRQ in the 3 age groups. In the younger group, gastrointestinal and respiratory factor scores had strong or weak positive correlations with HPs and normalized HAs (*r*: 0.257–0.317), while tinnitus–lump factor scores had a weak negative correlation with the 10th HP (*r* = −0.290). Stomach upset, edema–weight gain, and eating response factor scores of the FRQ had strong or weak positive correlations with HPs (*r*: 0.267–0.370) and normalized HAs (*r*: 0.263–0.300). Pain–bruising factor scores of the BSQ had a strong positive correlation with the sixth HP (*r* = 0.312). In middle age group, psychological factor scores had a strong positive correlation with the 12th HP (*r* = 0.335), while respiratory factor scores had weak negative correlations with THD2 and AH2/AH1 (*r* = −0.274). Trauma factor scores of the BSQ had weak positive correlations with the fourth, fifth, sixth, and seventh HP indices (*r*: 0.278–0.292), and edema–weight gain factor scores of the FRQ had strong or weak positive correlations with the seventh, eighth, ninth, and 11th HP indices (*r*: 0.283–0.315). In the older group, psychological and respiratory factor scores of the PPQ had strong or weak negative correlations with normalized HAs (*r*: −0.294 to −0.410), while neurological and pain–fatigue factor scores had strong or weak positive correlations with normalized HAs (*r*: 0.263–0.319). Respiratory factor scores of the PPQ had strong or weak negative correlations with the first, second, third, fourth, fifth, sixth, and seventh HP indices (*r*: −0.270 to −0.440), while pain–fatigue factor scores had weak positive correlations with the 12th HP index (*r* = 0.282). However, none of the factor scores of the BSQ and FRQ showed significant correlations with HCs.

**Table 6:** Correlations between harmonic components and pathological pattern questionnaire scores in the younger group.

Scale	Subscale	Phlegm						Blood stasis			Food retention			
		PP 1	PP 2	PP3	PP 4	PP 5	PP 6	BS 1	BS 2	BS 3	FR 1	FR 2	FR 3	FR 4
Total harmonic distortion	THD_2_	0.050	−0.007	−0.215	0.056	−0.057	−0.033	−0.087	−0.076	−0.059	−0.090	0.033	−0.166	−0.059
	THD_3_	0.054	−0.023	−0.219	0.031	−0.058	−0.018	−0.083	−0.100	−0.077	−0.083	0.036	−0.172	−0.068
	THD_4_	0.055	−0.020	−0.218	0.032	−0.057	−0.017	−0.080	−0.097	−0.077	−0.079	0.038	−0.175	−0.065
	THD_5_	0.055	−0.017	−0.217	0.037	−0.054	−0.015	−0.076	−0.093	−0.075	−0.075	0.043	−0.177	−0.063
	THD_6_	0.055	−0.016	−0.216	0.039	−0.052	−0.014	−0.074	−0.093	−0.075	−0.074	0.045	−0.176	−0.061
	THD_7_	0.055	−0.016	−0.216	0.040	−0.052	−0.014	−0.074	−0.092	−0.075	−0.074	0.045	−0.176	−0.061
	THD_8_	0.055	−0.016	−0.216	0.040	−0.052	−0.014	−0.073	−0.093	−0.075	−0.074	0.045	−0.176	−0.061
	THD_9_	0.055	−0.016	−0.216	0.040	−0.052	−0.014	−0.073	−0.093	−0.075	−0.074	0.045	−0.176	−0.061
	THD_10_	0.063	−0.010	−0.213	0.054	−0.062	−0.007	−0.060	−0.097	−0.077	−0.065	0.045	−0.163	−0.060
	THD_11_	0.064	−0.009	−0.213	0.056	−0.064	−0.006	−0.057	−0.098	−0.077	−0.064	0.045	−0.161	−0.060
	THD_12_	0.088	0.012	−0.199	0.101	−0.095	0.015	−0.011	−0.113	−0.082	−0.034	0.045	−0.115	−0.055
Harmonic frequency	Fundamental frequency	−0.236	−0.010	−0.005	−0.178	0.054	−0.112	−0.250	0.116	0.124	**−0.260** *	−0.051	−0.367**	−0.073
Harmonic power	1st HP	0.059	−0.058	0.148	0.004	−0.192	−0.011	0.057	−0.166	0.038	0.071	0.002	0.165	0.089
	2nd HP	0.076	−0.053	0.015	0.032	−0.191	−0.026	0.003	−0.179	0.002	0.014	0.018	0.055	0.045
	3rd HP	0.080	−0.105	−0.022	−0.066	−0.177	0.041	0.004	−0.247	−0.084	0.035	0.016	0.010	−0.004
	4th HP	0.091	0.061	0.072	0.008	−0.145	0.039	0.118	−0.106	−0.003	0.152	0.069	−0.049	0.112
	5th HP	0.074	0.074	0.108	0.089	−0.106	0.130	0.208	−0.023	0.019	**0.268** *	0.204	−0.063	0.086
	6th HP	0.139	0.046	0.136	0.119	−0.009	0.167	**0.312** *	−0.107	−0.052	0.338**	0.210	0.064	0.075
	7th HP	0.150	−0.087	0.185	0.179	−0.104	0.036	0.155	−0.108	−0.055	0.233	0.111	0.094	0.013
	8th HP	0.226	−0.102	0.186	0.131	−0.224	−0.042	0.065	−0.122	−0.048	0.219	0.197	0.053	−0.107
	9th HP	0.209	−0.111	0.186	0.055	−0.253	0.045	0.049	−0.132	−0.051	0.156	0.054	0.166	0.047
	10th HP	**0.257** *	−0.145	0.181	**0.257** *	**−0.290** *	0.090	0.090	−0.060	−0.012	0.147	0.026	0.370**	−0.011
	11th HP	0.209	−0.030	0.149	**0.261** *	−0.254	0.041	0.038	−0.018	−0.077	0.179	−0.027	**0.301** *	0.002
	12th HP	0.349**	0.059	0.136	0.222	−0.241	0.045	0.158	0.001	−0.039	**0.267** *	0.015	0.255	0.130
Normalized harmonic amplitude	AH_2_/AH_1_	−0.025	0.028	−0.116	0.073	0.011	−0.056	−0.068	0.030	−0.061	−0.123	0.016	−0.238	0.049
	AH_3_/AH_1_	0.048	−0.032	−0.126	−0.031	0.007	0.043	−0.006	−0.054	−0.077	0.003	0.116	−0.231	−0.001
	AH_4_/AH_1_	0.062	0.149	−0.025	0.045	0.034	0.023	0.064	0.182	−0.020	0.143	0.103	−0.253	0.085
	AH_5_/AH_1_	0.082	0.143	−0.002	0.163	0.047	0.143	0.120	0.224	0.071	0.193	0.177	−0.222	0.049
	AH_6_/AH_1_	0.090	−0.038	0.046	0.237	0.091	0.227	0.254	0.035	−0.117	0.250	**0.300** *	0.093	−0.044
	AH_7_/AH_1_	0.225	−0.075	0.094	**0.317** *	−0.125	0.104	0.125	−0.007	−0.115	0.189	0.076	0.153	−0.037
	AH_8_/AH_1_	0.205	0.037	0.094	**0.314** *	−0.225	0.168	0.175	−0.037	−0.106	0.243	0.090	0.158	−0.143
	AH_9_/AH_1_	0.216	0.082	0.065	**0.262** *	−0.225	0.096	0.235	−0.116	−0.061	0.187	−0.001	**0.269** *	−0.012
	AH_10_/AH_1_	0.155	0.127	0.059	**0.278** *	−0.197	0.128	**0.278** *	−0.099	−0.039	0.173	0.004	**0.263** *	0.024
	AH_11_/AH_1_	0.156	0.127	0.059	**0.279** *	−0.198	0.128	**0.278** *	−0.098	−0.039	0.173	0.004	**0.263** *	0.024
	AH_12_/AH_1_	0.155	0.127	0.059	**0.278** *	−0.197	0.128	**0.278** *	−0.099	−0.039	0.172	0.004	**0.263** *	0.025

Bold letters indicated significant *P* value.

AH = amplitude of harmonics, BS = blood stasis, FR = food retention, HP = harmonic power, PP = phlegm pattern, THD = total harmonic distortion, VAI = vascular aging index.

* *P* < .05.

**P < .01.

**Table 7:** Correlations between harmonic components and pathological pattern questionnaire scores in the middle age group.

Scale	Subscale	Phlegm pattern						Blood stasis pattern			Food retention pattern			
		PP 1	PP 2	PP3	PP 4	PP 5	PP 6	BS 1	BS 2	BS 3	FR 1	FR 2	FR 3	FR 4
Total harmonic distortion	THD_2_	−0.105	−0.152	<0.001	**−0.274** *	−0.165	0.042	−0.190	−0.097	−0.060	−0.146	−0.115	−0.166	−0.162
	THD_3_	−0.094	−0.171	−0.005	−0.257	−0.124	0.014	−0.201	−0.065	−0.031	−0.138	−0.124	−0.154	−0.156
	THD_4_	−0.099	−0.167	−0.006	−0.256	−0.119	0.009	−0.202	−0.062	−0.026	−0.145	−0.116	−0.152	−0.153
	THD_5_	−0.098	−0.165	−0.007	−0.254	−0.114	0.007	−0.201	−0.062	−0.021	−0.147	−0.112	−0.151	−0.152
	THD_6_	−0.097	−0.164	−0.008	−0.254	−0.113	0.008	−0.201	−0.061	−0.021	−0.147	−0.111	−0.150	−0.152
	THD_7_	−0.097	−0.164	−0.008	−0.254	−0.112	0.008	−0.201	−0.061	−0.021	−0.147	−0.111	−0.150	−0.151
	THD_8_	−0.097	−0.164	−0.008	−0.254	−0.112	0.008	−0.201	−0.062	−0.020	−0.147	−0.110	−0.150	−0.151
	THD_9_	−0.097	−0.164	−0.008	−0.254	−0.112	0.008	−0.201	−0.062	−0.020	−0.147	−0.110	−0.150	−0.151
	THD_10_	−0.097	−0.164	−0.008	−0.254	−0.112	0.008	−0.201	−0.062	−0.020	−0.147	−0.110	−0.150	−0.151
	THD_11_	−0.097	−0.164	−0.008	−0.254	−0.112	0.008	−0.201	−0.062	−0.020	−0.147	−0.110	−0.150	−0.151
	THD_12_	−0.097	−0.164	−0.008	−0.254	−0.112	0.008	−0.201	−0.062	−0.021	−0.147	−0.110	−0.151	−0.151
Harmonic frequency	Fundamental frequency	−0.067	0.108	−0.002	−0.110	−0.170	0.060	0.016	−0.258	0.043	−0.122	0.150	−0.058	−0.026
Harmonic power	1st HP	0.101	0.156	0.239	0.160	0.003	−0.226	0.023	0.066	0.251	0.170	0.129	−0.107	−0.012
	2nd HP	0.064	0.100	0.226	0.067	−0.048	−0.200	−0.037	0.033	0.219	0.116	0.086	−0.152	−0.061
	3rd HP	0.073	0.025	0.197	0.087	0.054	−0.248	−0.065	0.118	0.261	0.117	0.059	−0.115	−0.066
	4th HP	−0.028	0.127	0.194	0.024	0.059	−0.235	−0.085	0.080	**0.285** *	−0.027	0.187	−0.085	−0.100
	5th HP	0.121	0.184	0.199	0.083	0.092	−0.214	0.024	0.078	**0.292** *	0.032	0.218	−0.032	−0.055
	6th HP	0.108	0.150	0.192	0.055	0.206	−0.162	0.032	0.141	**0.297** *	0.007	0.260	0.011	−0.042
	7th HP	0.031	0.150	0.118	0.011	0.180	−0.100	−0.040	0.115	**0.278** *	0.007	**0.315** *	0.006	−0.109
	8th HP	−0.014	0.161	0.097	0.075	0.056	−0.023	0.006	0.019	0.181	0.035	**0.293** *	−0.047	−0.112
	9th HP	0.041	0.089	0.042	0.053	0.111	−0.112	−0.043	0.095	0.202	0.031	**0.283** *	−0.021	−0.074
	10th HP	<0.001	0.102	0.046	−0.004	0.162	−0.214	−0.161	0.048	0.241	0.004	0.235	−0.006	−0.132
	11th HP	−0.037	0.118	0.064	0.110	0.118	−0.229	−0.003	0.117	0.010	0.001	**0.303** *	−0.059	0.116
	12th HP	−0.008	**0.335** *	−0.017	0.188	0.073	−0.125	0.122	0.062	0.175	0.146	0.260	0.034	−0.025
Normalized harmonic amplitude	AH_2_/AH_1_	−0.067	−0.159	−0.095	**−0.274** *	−0.147	0.032	−0.196	−0.095	−0.054	−0.139	−0.107	−0.156	−0.156
	AH_3_/AH_1_	0.027	−0.228	−0.109	−0.175	0.063	−0.068	−0.197	0.045	0.077	−0.024	−0.177	−0.085	−0.097
	AH_4_/AH_1_	−0.143	−0.031	−0.103	−0.193	0.015	−0.030	−0.169	0.008	0.061	−0.240	0.079	−0.081	−0.008
	AH_5_/AH_1_	0.040	0.008	−0.097	−0.139	0.072	−0.058	−0.131	0.004	0.133	−0.168	0.065	−0.002	−0.071
	AH_6_/AH_1_	0.128	0.019	−0.130	−0.216	0.135	0.059	−0.173	0.050	0.026	−0.120	0.028	−0.043	−0.107
	AH_7_/AH_1_	0.018	−0.018	−0.166	−0.253	0.112	0.059	−0.176	−0.059	0.021	−0.171	0.111	−0.091	0.007
	AH_8_/AH_1_	−0.033	0.048	−0.111	−0.084	0.130	−0.024	−0.019	−0.080	0.072	−0.105	0.229	−0.053	0.135
	AH_9_/AH_1_	−0.045	−0.031	−0.182	−0.064	0.144	0.022	−0.028	−0.092	0.073	−0.116	0.164	−0.033	0.160
	AH_10_/AH_1_	−0.176	−0.046	−0.067	−0.091	0.213	−0.020	−0.102	−0.053	0.137	−0.148	0.168	−0.048	0.081
	AH_11_/AH_1_	−0.012	−0.006	−0.165	−0.052	0.100	−0.026	−0.038	−0.054	−0.010	0.004	0.012	−0.085	0.138
	AH_12_/AH_1_	0.067	0.121	−0.174	−0.049	0.072	0.026	0.045	−0.009	−0.007	0.130	−0.038	−0.087	0.165

Bold letters indicated significant *P* value.

AH = amplitude of harmonics, HP = harmonic power, THD = total harmonic distortion, VAI = vascular aging index.

* *P* < .05.

**Table 8:** Correlations between harmonic components and pathological pattern questionnaire scores in the older women.

Scale	Subscale	Phlegm pattern						Blood stasis pattern			Food retention pattern			
		PP 1	PP 2	PP3	PP 4	PP 5	PP 6	BS 1	BS 2	BS 3	FR 1	FR 2	FR 3	FR 4
Total harmonic distortion	THD_2_	0.053	−0.128	−0.081	−0.223	0.053	0.139	−0.114	0.076	−0.115	0.054	−0.018	0.026	−0.026
	THD_3_	0.059	−0.099	−0.071	−0.248	0.066	0.106	−0.096	0.059	−0.118	0.062	−0.001	0.029	−0.041
	THD_4_	0.062	−0.098	−0.065	−0.251	0.070	0.103	−0.092	0.058	−0.115	0.066	0.004	0.030	−0.041
	THD_5_	0.062	−0.096	−0.063	−0.252	0.072	0.102	−0.089	0.058	−0.114	0.067	0.007	0.030	−0.040
	THD_6_	0.062	−0.097	−0.062	−0.252	0.072	0.103	−0.088	0.058	−0.114	0.067	0.008	0.030	−0.039
	THD_7_	0.062	−0.097	−0.062	−0.252	0.072	0.104	−0.087	0.058	−0.114	0.067	0.009	0.030	−0.039
	THD_8_	0.062	−0.097	−0.062	−0.252	0.073	0.104	−0.087	0.058	−0.114	0.067	0.009	0.030	−0.039
	THD_9_	0.062	−0.097	−0.062	−0.252	0.073	0.104	−0.087	0.058	−0.114	0.067	0.009	0.030	−0.039
	THD_10_	0.062	−0.098	−0.061	−0.252	0.072	0.105	−0.087	0.058	−0.113	0.067	0.009	0.030	−0.039
	THD_11_	0.062	−0.099	−0.061	−0.252	0.072	0.105	−0.087	0.058	−0.113	0.067	0.009	0.030	−0.039
	THD_12_	0.062	−0.099	−0.060	−0.251	0.072	0.105	−0.087	0.058	−0.113	0.067	0.009	0.030	−0.038
Harmonic frequency	Fundamental frequency	0.074	0.124	−0.164	−0.009	−0.002	−0.171	−0.011	0.091	−0.143	0.051	−0.173	0.144	−0.041
Harmonic power	1st HP	−0.116	0.133	−0.235	**−0.270** *	−0.027	−0.065	−0.168	−0.087	0.035	−0.191	−0.025	−0.010	−0.088
	2nd HP	−0.074	0.057	−0.229	**−0.318** *	<0.001	0.005	−0.188	−0.041	−0.019	−0.135	−0.028	0.003	−0.084
	3rd HP	−0.036	0.156	−0.181	**−0.372** **	0.057	−0.096	−0.097	−0.102	−0.034	−0.051	0.035	−0.002	−0.136
	4th HP	0.066	0.125	−0.024	**−0.440** **	0.100	−0.061	0.003	−0.044	−0.033	0.113	0.113	−0.022	−0.128
	5th HP	−0.008	0.099	−0.046	**−0.348** **	0.016	−0.030	−0.043	−0.090	−0.026	−0.013	0.129	0.006	−0.125
	6th HP	−0.020	0.042	−0.091	**−0.318** *	−0.068	0.029	−0.053	−0.159	−0.079	−0.098	0.068	0.035	−0.118
	7th HP	0.023	0.033	−0.097	**−0.376** **	−0.031	0.055	−0.041	−0.095	−0.033	−0.040	0.106	0.027	−0.139
	8th HP	0.017	−0.071	−0.023	−0.256	−0.023	0.133	−0.054	−0.018	0.024	0.034	0.008	0.050	−0.084
	9th HP	−0.012	−0.029	−0.160	−0.253	0.037	0.127	−0.093	−0.013	0.029	−0.106	0.091	0.075	−0.044
	10th HP	−0.068	−0.121	−0.052	−0.145	0.021	0.136	−0.008	−0.066	0.019	−0.101	0.036	0.079	0.020
	11th HP	−0.030	−0.142	0.018	0.093	0.047	0.115	−0.003	0.031	0.069	−0.079	0.237	0.224	−0.099
	12th HP	0.104	−0.211	0.203	−0.074	0.050	**0.282** *	0.037	0.154	0.002	0.087	0.053	0.111	−0.028
Normalized harmonic amplitude	AH_2_/AH_1_	0.081	−0.247	0.064	−0.181	−0.069	0.236	−0.141	0.085	−0.121	0.052	−0.046	0.039	−0.046
	AH_3_/AH_1_	0.110	0.095	0.069	**−0.294** *	0.010	−0.134	0.035	−0.082	−0.078	0.053	0.083	0.074	−0.150
	AH_4_/AH_1_	0.218	−0.126	**0.272** *	−0.211	0.088	−0.017	0.081	0.027	0.051	0.166	0.155	0.125	−0.048
	AH_5_/AH_1_	0.083	−0.107	0.182	−0.174	0.062	0.039	0.067	0.051	−0.002	0.070	0.164	0.066	<0.001
	AH_6_/AH_1_	0.038	**−0.296** *	0.227	−0.029	0.018	**0.268** *	0.069	0.037	−0.018	−0.015	0.095	0.040	0.092
	AH_7_/AH_1_	0.081	**−0.356** **	**0.279** *	−0.023	0.062	**0.263** *	0.053	0.097	0.039	0.049	0.080	0.053	0.094
	AH_8_/AH_1_	0.034	**−0.410** **	**0.264** *	0.041	0.010	**0.285** *	−0.041	0.134	0.050	0.032	0.005	0.019	0.099
	AH_9_/AH_1_	0.030	**−0.383** **	0.230	0.044	0.008	**0.306** *	−0.030	0.133	0.018	0.004	0.019	0.017	0.107
	AH_10_/AH_1_	0.017	**−0.377** **	0.223	0.047	−0.064	**0.318** *	−0.094	0.077	−0.001	−0.015	−0.025	0.013	0.075
	AH_11_/AH_1_	0.020	**−0.376** **	0.224	0.048	−0.063	**0.318** *	−0.093	0.079	−0.002	−0.015	−0.023	0.016	0.073
	AH_12_/AH_1_	0.019	**−0.377** **	0.223	0.048	−0.065	**0.319** *	−0.097	0.079	−0.002	−0.015	−0.026	0.014	0.073

Bold letters indicated significant *P* value.

AH = amplitude of harmonics, HP = harmonic power, THD = total harmonic distortion, VAI = vascular aging index.

* *P* < .05.

** *P* < .01.

## 4. Discussion

In this study, we examined whether HCs of PTG may serve as an arterial stiffness marker by comparing the 3 measurement units of HCs with the SDPTG indices. Furthermore, we examined the relationship between HCs of PTG and PP questionnaire scores, referring to the dysfunction of internal organs. In addition, we examined whether the relationships between indices of HCs, SDPTG, and questionnaire scores were general or local. The main finding of this study is that HCs indices had an age effect and were related to SDPTG indices, indicating that HCs could serve as an arterial stiffness marker; this is consistent with the findings of a previous study.^[21]^ Another finding of this study was that the indices of HCs were generally related to factor scores of PPQ, BSQ, and FRQ.

The age effect of HCs and relationship between HC and SDPTG indices were reported in previous studies. However, previous studies examined the aging effect using only normalized HA. This study found that not only normalized HA but also HP and THD indices had aging effects. Specially, all THDs had an aging effect, although their standardized values were lower than those of normalized amplitudes. The increases in HCs after THD_5_ may be too small to affect the THDs, and standardized *β* values of the THDs after THD_5_ may have changed a little. In terms of normalized harmonic amplitude, there was decrease in AH_2_/AH_1_, AH_3_/AH_1_, AH_4_/AH_1_, and AH_5_/AH_1_ with aging, which is consistent with that reported in a previous study where the second, third, fourth, fifth, and sixth HA decreased with aging.^[21]^ Only 4 HAs among 11 normalized HAs had aging effects, similar to the findings of a previous study.^[21]^ It is unclear why the aging effect reflected up till the fifth or sixth normalized HAs. One reason is that there may be 2 groups among all HCs – one group with higher HP and the other group with lower HP. According to this premise, the former may have been associated with reflective components of PTG, while the latter may not. Among all HPs, only the fourth HP had an aging effect. Our study suggests that the aging effect of THD and normalized HA may be stronger than that of HP.

As HCs of PTG had an aging effect, we divided the subjects into 3 groups (younger, middle age, and older groups) and examined the relationship between HCs and SDPTG indices in 3 age groups. Regarding the relationship between normalized HA and SDPTG indices, AH_3_/AH_1_, AH_4_/AH_1_, and AH_5_/AH_1_ had strong negative correlations with *b/a*, while had strong or weak correlations with *d/a*. These relationships were consistent with those reported in a previous study.^[21]^ Moreover, in this study, the relationships were prominent in middle age and older groups and not in younger group. Together with normalized HAs, all THDs had strong or weak negative correlations with *b/a* and weak positive correlations with *d/a* in the middle age group. In the older group, the fourth, seventh, and eighth HPs had strong or weak negative correlations with *b/a* and positive correlations with *d/a*. From the SDPTG point of view, *b* wave corresponds to the early stage of ventricular ejection, and increased *b/a* denotes increased large artery stiffness, while *d* wave corresponds to the late stage of ventricular ejection, and decreased *d/a* denotes increased peripheral artery resistance.^[7]^ In the younger group, there was no significant correlation between HCs and SDPTG indices except VAI, *d/a*, and AH_2_/AH_1,_ indicating that the effect of age may not linearly increase with aging. For women, HCs, including THD, normalized HA, and HP appear to remain stable until the early 30s, and then change after the mid-30s. We hypothesized that THD may serve as an impediment index of blood circulation, similar to an electrical circuit, and the study result showed that it was related to *b/a* and *d/a* of the SDPTG. Therefore, the clinical use of THD as an arterial stiffness marker deserves further studies, including studies evaluating why its application was localized to the middle age group. In summary, our study results suggest that in the middle age and older groups, increased THD, HP, and normalized HA may reflect increased arterial stiffness and peripheral resistance, similar to SDPTG indices.

Another main finding of our study was that clinical severity of phlegm, BS, and FR patterns estimated using questionnaire scores were related to HC indices of PTG. Moreover, these relationships were different between age groups. In the younger group, 3 factors score of the PPQ (gastrointestinal, respiratory, and tinnitus–lump factors), one factor score of the BSQ (pain–bruising factor), and 3 factors scores of the FRQ (stomach upset, edema–weight gain, and food allergy factors) had strong or weak positive correlations with HP and normalized HA. THDs had correlations with none of the factor scores of the PP questionnaires, indicating that in the younger group, increased power and amplitude of HCs reflected aggravated phlegm, BS, and FR patterns. However, the relationship between HCs and PPs was weaker in the middle age group than in the young group; that is, except for THD_2_ and AH_2_/AH_1_, the relationship between HCs and PPs was limited to the fourth to 11th HPs. These HPs had strong or weak positive correlations with the scores of BSQ trauma factor, and the scores of FRQ edema–weight gain factor. In the older group, HCs had positive or negative correlations limitedly with phlegm patterns; that is, the first, second, third, fourth, fifth, sixth, and seventh HPs and normalized HAs from AH_6_/AH_1_ to AH_12_/AH_1_ had strong or weak negative correlations with psychological and respiratory factors scores of the PPQ, while they had strong or weak positive correlations with neurological and pain–fatigue factor scores of the PPQ. This meant that the phlegm pattern might be related to an increase or decrease in power or amplitude of HCs in the older group.

Observing the relationships between HCs and SDPTG indices and between HCs and PP scores simultaneously, 2 characteristics were confirmed. First, increased arterial stiffness was positively correlated with decreased THD, HP, or normalized HA, while progressive PP were positively correlated with increased HCs. Second, these relationships showed a mixture of one-to-one single relationships (e.g. relationship between *d/a* and AH_2_/AH_1_ in the younger group or relationship between FR2 scores and AH_6_/AH_1_ in younger group) and group relationships with several others (e.g. relationship between *b/a* and all THDs in the middle age group or relationship between PP6 scores and normal HAs from the sixth to the 12th components in the older group). HCs decrease with aging or increased arterial stiffness.^[1,14,21]^ Combining the concept of THD and HP with normalized HA, our study supports the previous study results of negative correlations between HCs and arterial stiffness. However, except for the aging effect and arterial stiffness marker of HCs, the changes in HCs reported in the previous study of other physiological or pathological conditions have not been consistent. For example, ligation of the main artery and atopic dermatitis was associated with decreased HCs,^[17,20]^ while ingesting food and open-angle glaucoma were associated with increased HCs.^[29,30]^ Positive correlations between PP scores and HCs implied that increased phlegm, BS, and FS patterns may be associated with increased HCs, like ingesting food or glaucoma condition. A study speculated that the increase in HCs may result from lowered rigidity of arterial wall.^[28]^ If this premise is right, reflective components may have increased on the condition of phlegm-inducing neurological and pain–fatigue problems, while reflective components may have decreased on the condition of phlegm-inducing psychological and respiratory problems in the older group. Therefore, further studies are necessary for elucidating the mechanism of changes in HCs using the progression of PPs. Further, a mixture of sporadic and general relationships between HCs and PP scores were inconsistent with Wang’s “frequency–organ relation,” where one HC was related to one internal organ.^[19]^ One possibility is that PPs gradually change and may be reflected on HCs more broadly than other pathological conditions.

This study has some limitations. This chart review study included only female subjects. Therefore, examination of sex differences in the relationship between HC indices and PPs is needed in future studies. The female subjects were outpatients with gynaecological problems and these problems may have affected the characteristics of HC indices. To address this concern, it is necessary to examine HC indices of PTG in a normal population. This study limited PPs to etiological factors impeding the circulation of blood or fluid. Therefore, a study of the relationship between deficiency PPs such as qi deficiency and HCs are needed in the future. Some confounding factors such as blood pressure and body mass index were reported to affect arterial stiffness.^[7,31]^ Therefore, the effect of confounding factors on relationship between HCs and pathological patterns are needed.

Conclusively, the study findings suggest that THD, HP, and normalized HA had an aging effect and reflected arterial stiffness. HCs increased on the progressive condition of phlegm, BS, and FR patterns, and the increase in HCs was different among the younger, middle age, and older groups. Further studies are needed to address the limitation of sex differences, normal participants, confounding factors and deficiency PPs.

## 5. Conclusion

In this study, aging effect of HCs and the relationship between HC and SDPTG indices and scores of 3 the PP questionnaires, PPQ, BSQ, and FRQ, were examined in 173 women with gynaecological problems. Three HCs – THD, HP, and normalized HA – were calculated to examine which indices were more indicative of arterial stiffness or the clinical severity of phlegm, BS, and FR patterns. This study revealed that HCs had an aging effect and reflected arterial stiffness. HCs also increased on the progressive condition of phlegm, BS, and FR patterns. In conclusion, HCs may serve as an arterial stiffness marker, and may be partially related to phlegm, BS, and FR patterns. Aging effect needs to be considered when utilizing HCs as an indicator of phlegm, BS, and FR patterns. Further studies are needed to address the limitation of sex differences, normal participants, confounding factors and deficiency PPs.

## Author contributions

**Conceptualization:** Young-Jae Park.

**Data curation:** Jin-Moo Lee, Ka-Hye Choi.

**Formal analysis:** Young-Jae Park, Ka-Hye Choi.

**Investigation:** Jin-Moo Lee.

**Writing – review & editing:** Young-Jae Park.
